# Transcriptome analysis reveals the key pathways and candidate genes involved in salt stress responses in *Cymbidium ensifolium* leaves

**DOI:** 10.1186/s12870-023-04050-z

**Published:** 2023-02-01

**Authors:** Xiang Li, Lanlan Liu, Shixian Sun, Yanmei Li, Lu Jia, Shili Ye, Yanxuan Yu, Komivi Dossa, Yunpeng Luan

**Affiliations:** 1 The First Affiliated Hospital of Yunnan University of Traditional Chinese Medicine, 650021 Kunming, China; 2 Key Laboratory for Forest Resources Conservation and Utilization in the Southwest Mountains of China, Ministry of Education, Southwest Forestry University, 650224 Kunming, China; 3 Yunnan Key Laboratory of Plateau Wetland Conservation, Restoration and Ecological Services, Southwest Forestry University, 650224 Kunming, China; 4 Department of Life Technology Teaching and Research, School of Life Science, Southwest Forestry University, 650224 Kunming, China; 5 Faculty of Mathematics and Physics, Southwest Forestry University, 650224 Kunming, China; 6 CIRAD, UMR AGAP Institute, F-34398 Montpellier, France

**Keywords:** ABA signaling, Ca^2+^ signaling, Ion balance, Na^+^/K^+^ exchangers, Na^+^/H^+^ antiporters, Salinity stress sensing

## Abstract

**Background:**

*Cymbidium ensifolium* L. is known for its ornamental value and is frequently used in cosmetics. Information about the salt stress response of *C. ensifolium* is scarce. In this study, we reported the physiological and transcriptomic responses of *C. ensifolium* leaves under the influence of 100 mM NaCl stress for 48 (T48) and 96 (T96) hours.

**Results:**

Leaf Na^+^ content, activities of the antioxidant enzymes i.e., superoxide dismutase, glutathione S-transferase, and ascorbate peroxidase, and malondialdehyde content were increased in salt-stressed leaves of *C. ensifolium*. Transcriptome analysis revealed that a relatively high number of genes were differentially expressed in CKvsT48 (17,249) compared to CKvsT96 (5,376). Several genes related to salt stress sensing (calcium signaling, stomata closure, cell-wall remodeling, and ROS scavenging), ion balance (Na^+^ and H^+^), ion homeostasis (Na^+^/K^+^ ratios), and phytohormone signaling (abscisic acid and brassinosteroid) were differentially expressed in CKvsT48, CKvsT96, and T48vsT96. In general, the expression of genes enriched in these pathways was increased in T48 compared to CK while reduced in T96 compared to T48. Transcription factors (TFs) belonging to more than 70 families were differentially expressed; the major families of differentially expressed TFs included bHLH, NAC, MYB, WRKY, MYB-related, and C3H. A Myb-like gene (*CenREV3*) was further characterized by overexpressing it in *Arabidopsis thaliana*. *CenREV3’s* expression was decreased with the prolongation of salt stress. As a result, the *CenREV3-*overexpression lines showed reduced root length, germination %, and survival % suggesting that this TF is a negative regulator of salt stress tolerance.

**Conclusion:**

These results provide the basis for future studies to explore the salt stress response-related pathways in *C. ensifolium.*

**Supplementary Information:**

The online version contains supplementary material available at 10.1186/s12870-023-04050-z.

## Background

*Cymbidium ensifolium* L., also known as Sword-Leaf Cymbidium, belongs to *Orchidaceae*, one of the largest and most evolved monocot families [1]. It is naturally distributed in the tropical and subtropical areas of Borneo, China, Indonesia, Japan, New Guinea, and the Philippines. It has been cultivated as an ornamental plant for 2000 years [2]. Nowadays, it has gained an important status as a commercial floristic plant and is valued for cut flowers, hanging baskets, and potted plants. It is also used in medicine, cosmetics, and food [3]. It is usually propagated by new shoots and can flower throughout the year [4]. Recently, the use of *Cymbidium* products has been increasingly appreciated by consumers, especially during the Spring festivals in China. Since China is the largest consumer of orchid cut flowers, research on various aspects of growth, development, and biotic and abiotic stress responses is needed.

China faces severe abiotic stresses such as drought, cold, high temperature, salinity, and flooding. Nearly 4.88% of the available land area and nine million hectares of arable land in China are affected by salt [5]. The salinized soils are mainly located in the northwestern, northern, and northeastern regions of China [6], where *C. ensifolium* is mainly cultivated. The natural populations of *C. ensifolium* are declining and its habitats are now fragmented, which is why it is listed in the Red List of endangered species [7]. Its habitat can be expanded to other areas, but the use of already cultivated lands will affect farmers. The saline areas and the areas where only brackish water is available can be exploited [8]. In addition, the fragmented populations that are already declining due to salinity should be protected. In this context, a clear understanding of the salinity-response mechanisms in *C. ensifolium* is needed. However, relevant information is sparse. Moreover, there are few reports on the salt stress responses of some *Cymbidium* varieties/cultivars. For example, the *Cymbidium* hybrid “Twilight Moon” showed a significant decrease in fresh weight of the new protocorm-like bodies, indicating that *Cymbidium* is a strongly salt-sensitive plant [8]. In another study, the genes NPR1 and PR1 were identified and characterized to positively affect the biosynthesis of abscisic acid (ABA) and salinity tolerance in *Cymbidium* orchids [7]. The involvement of several osmolytes-related genes, transcription factors (TFs), antioxidant defense-related genes (e.g., glutathione S-transferase (GST) and ascorbate peroxidase (POD)), and ABA biosynthesis genes in salt stress tolerance was also found in other orchid species such as *Dendrobium officinale* [9]. The physiological responses of *Dendrobium* orchid showed a significant reduction in leaf and flower size, leaf dry weight, flower quality, and changes in Na^+^/K^+^ ratio [10]. Likewise, a study on *Spartina alterniflora* showed the mechanisms of salt-stress tolerance are complex [11]. Nevertheless, the responses of *C. ensifolium* to salt stress should be explored to develop appropriate strategies for expanding its natural distribution in salinized areas.

Plant growth and development are significantly affected by salt stress. High salt (NaCl) concentrations in the soil reduce water uptake by roots, which impairs nutrient uptake [12]. Such nutrient imbalance leads to osmotic stress and affects the plant at physiological, morphological, and molecular levels. The excess Na^+^ concentration in roots triggers ion imbalance, such as changes in intracellular Ca^2+^ level by modulating the expression of Ca^2+^ sensors and channels [13]. The changes in Ca^2+^ levels trigger the production of reactive oxygen species (ROS) and signal transduction, which in turn activates various adaptive processes to reduce the effects of the imposed stress [14]. For example, the activities of POD, GST, catalase (CAT), superoxide dismutase (SOD), and many other enzymes are increased to scavenge excessively accumulated ROS [15]. In addition to altering Ca^2+^ levels, the increased Na^+^ concentration also disrupts ion homeostasis (Na^+^/H^+^ and/or Na^+^/K^+^ exchange). To maintain the balance of ion concentrations, plants have evolved several molecular mechanisms such as activation/inactivation of Na^+^/H^+^ antiporters [16], K^+^ channels [17], calcineurin B-like proteins (CBLs), CBL-interacting protein kinases (CIPKs) [18], and calcium dependent protein kinases (CDPKs) [19]. The salt overly sensitive (SOS) regulatory pathway in plants also modulates the expressions/activities of Na^+^/H^+^ antiporters to maintain ionic balance [14, 20]. Plants are also able to activate osmolyte biosynthesis to overcome the salt-stress induced osmotic stress leading to changes in turgor pressure, plasma membrane, and cell wall. Osmolytes not only regulate osmotic potential but also act as signals, inducing the biosynthesis and signaling of phytohormones (e.g., ABA and brassinosteroids (BRs)), which in turn regulate stomata closure, cell wall structure, cell elongation, and cell division [21,22 and references therein].

Because natural populations of *C. ensifolium* are declining and habitats are fragmented, and few data are available on the responses to salt stress, we examined the physiological changes in *C. ensifolium* under 100 mM NaCl stress. We also report the transcriptomic responses of *C. ensifolium* leaves under 100 mM NaCl stress for 48 and 96 h. Based on the comparative transcriptome analysis and proteome analysis (data not included), we selected several salt-stress responsive genes, whose functions are currently under investigation in our laboratory. Among the selected genes, we report here a *CenREV3* gene *(REVEILLE),* which has a Myb-like DNA-binding domain and GO annotations for salt stress, transcription, and ABA response, etc. The REVs control various plant traits such as cell size, abiotic stress tolerance, and seed germination, and are also involved in of circadian-regulated processes [21–23]. Although the MYB TFs are associated with salt stress responses in many plant species, for example, a rice MYB TF *OsMYB91* showed a negative role in plant growth and salt stress tolerance in *Arabidopsis* [24], but there is no report on the role of *CenREV3* in salt stressed *C. ensifolium*. Therefore, we investigated the salt responses of the transgenic Arabidopsis lines overexpressing *CenREV3* and report its association with salt stress tolerance. Overall, this work provides new information on the dynamics of genes involved in salt sensing, ion balancing, osmotic homeostasis, ABA and BR signaling, and metabolic pathways involved in photosynthesis under salt stress.

## Results

### Physiological performance of C. ensifolium under salt stress

**Fig. 1:**
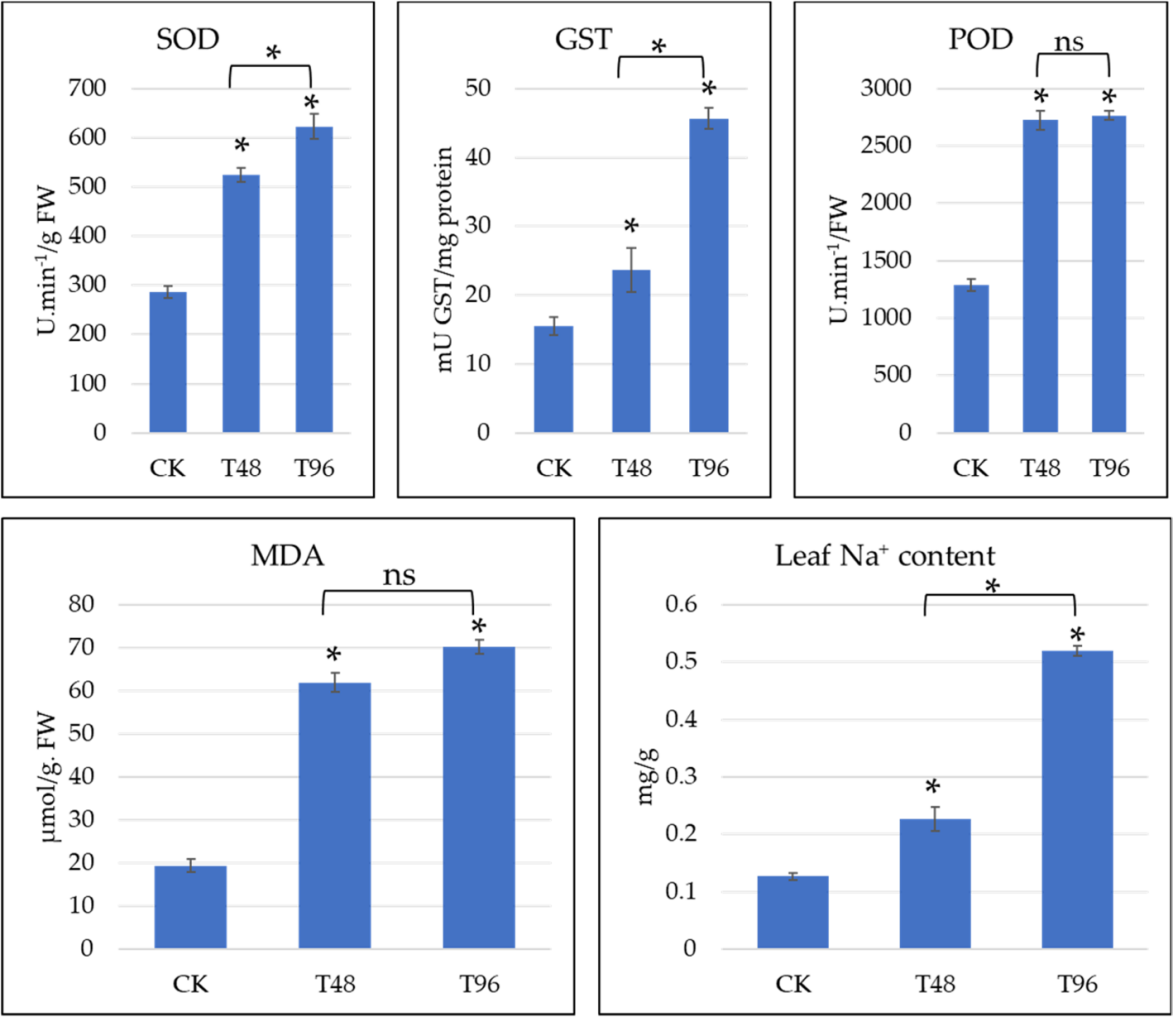
Physiological performance of *C. ensifolium* seedlings under salt stress. SOD; Superoxide dismutase, GST; Glutathione S-transferase, POD; Peroxidase, MDA; Malondialdehyde, and Leaf Na^+^ content. CK, T48, and T96 represent control (no NaCl treatment), 100 mM NaCl for 48, and 96 h, respectively. The values are the mean ± SD of three replicates (*p* < 0.05). The asterisk sign (*) represents significant differences

In this project, physiological performance (the activities of SOD, GST, and POD and MDA content) was studied in *C. ensifolium* leaves under the influence of 100 mM NaCl stress for 48 and 96 h. In general, the activities of these enzymes and the MDA content increased with time. Both SOD and GST activities increased significantly in T48 and T96 compared to CK. Their activities also increased significantly from T48 to T96. On the other hand, the activity of POD and the MDA content were significantly higher in T48 and T96 than in CK. However, the activity of POD and MDA content weren’t significantly different between T48 and T96. These results indicate that salt stress for 48 h has already caused a significant increase in the formation of ROS as well as lipid peroxidation. The relatively lower increase in the activities of SOD and POD as well as MDA content from T48 to T96 compared to the ones from CK to T48 and CK to 96, also support the proposition that most of the damage to the *C. ensifolium* leaves was caused by 48 h of salt stress, which either remained consistent or slowed down till 96 h. A significant increase in Na^+^ content (CK > T48 > T96) is also consistent with the above results and confirms the onset of salt stress (Fig. 1).

### Transcriptome profile of C. ensifolium leaves under drought stress

**Fig. 2:**
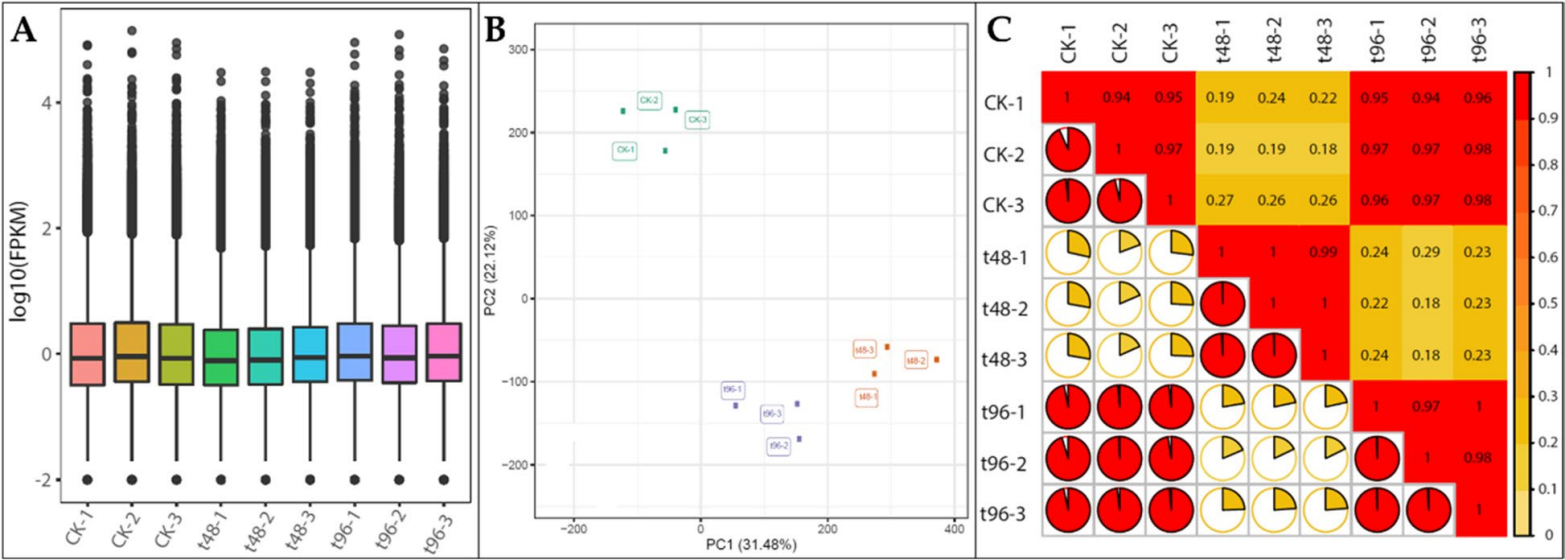
Transcriptome profile of *C. ensifolium* leaves under salt stress. **A** Overall distribution of sample gene expression, **B)** Principal Component Analysis of the expressed genes, and **C**) Pearson Correlation Coefficient (PCC) between CK, T48, and T96. The color bar in panel C represents a range of PCC

Sequencing of nine libraries produced an average of 47.95 million clean reads. The error rate and GC content were 0.02 and ~ 46%, respectively. The Q20% and Q30% were > 98% and > 95%, respectively (Supplementary Table 1). The overall distribution of gene expression was lower in T48 compared to T96 and CK (Fig. 2A). Grouping of the treatment replicates in Principal Component Analysis (PCA) shows that sampling was reliable. The PC1 and PC2 showed 31.48 and 22.12% variability, respectively (Fig. 2A). A relatively low Pearson correlation coefficient (PCC) was found between CK and T48 and T48 and T96, whereas a high PCC was observed between CK and T96 (Fig. 2C).

### Differential gene expression between salt treated and CK leaves

**Fig. 3:**
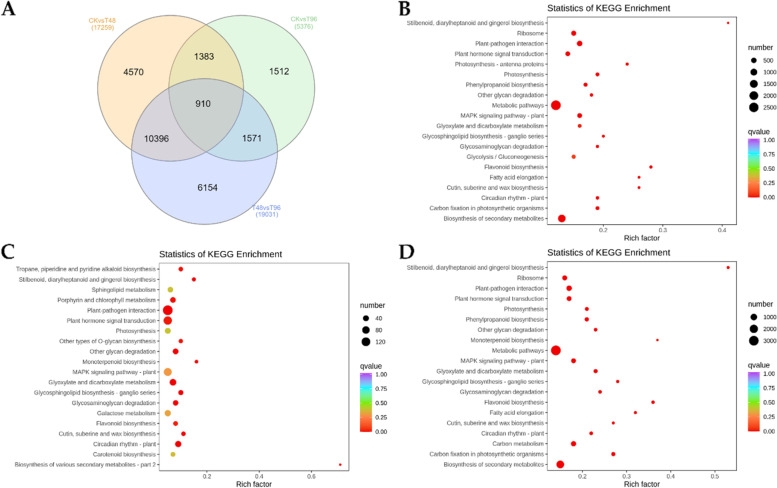
Summary of the number of differentially expressed genes and KEGG pathway enrichment analysis. **A** Venn diagram showing the number of DEGs in each treatment comparison. Statistics of enrichment of DEGs in KEGG pathways in **B**) CKvsT48, **C)** CKvsT96, and **D**) T48vsT96

Based on the screening criteria, 17,249, 5,376, and 19,031 differentially expressed genes (DEGs) were identified in CKvsT48, CKvsT96, and T48vsT96, respectively (Fig. 3A). A relatively higher number of DEGs in CKvsT48 indicates that salt stress for 48 h causes higher transcriptional activity in *C. ensifolium* leaves compared to 96 h, confirming the results of antioxidant enzyme activities and MDA content (Fig. 1). Of all DEGs, 57.42% could be annotated in at least one database (Supplementary Fig. 1). The DEGs in CKvsT48 were enriched in flavonoid biosynthesis, MAPK signaling-plant, stilbenoid, diarylheptanoid, and gingerol biosynthesis, photosynthesis-antenna proteins, carbon fixation, and photosynthesis pathways (Fig. 3B). In addition to these pathways, DEGs in CKvsT96, as well as T48vsT96, were also enriched in glycosphingolipid biosynthesis, cutin, suberin and wax biosynthesis, and monoterpenoid biosynthesis pathways (Fig. 3C-D).

### Expression changes in salt stress sensing genes

As the Na^+^ contents of leaves increased significantly over time (Fig. 1), we explored the expression changes in key genes/pathways related to signaling, salt signaling responses i.e., stomata closure and cell wall modification, and ROS scavenging.

### *Ca*^*2*+^*signaling-related expression changes between salt treated and control leaves*

**Fig. 4:**
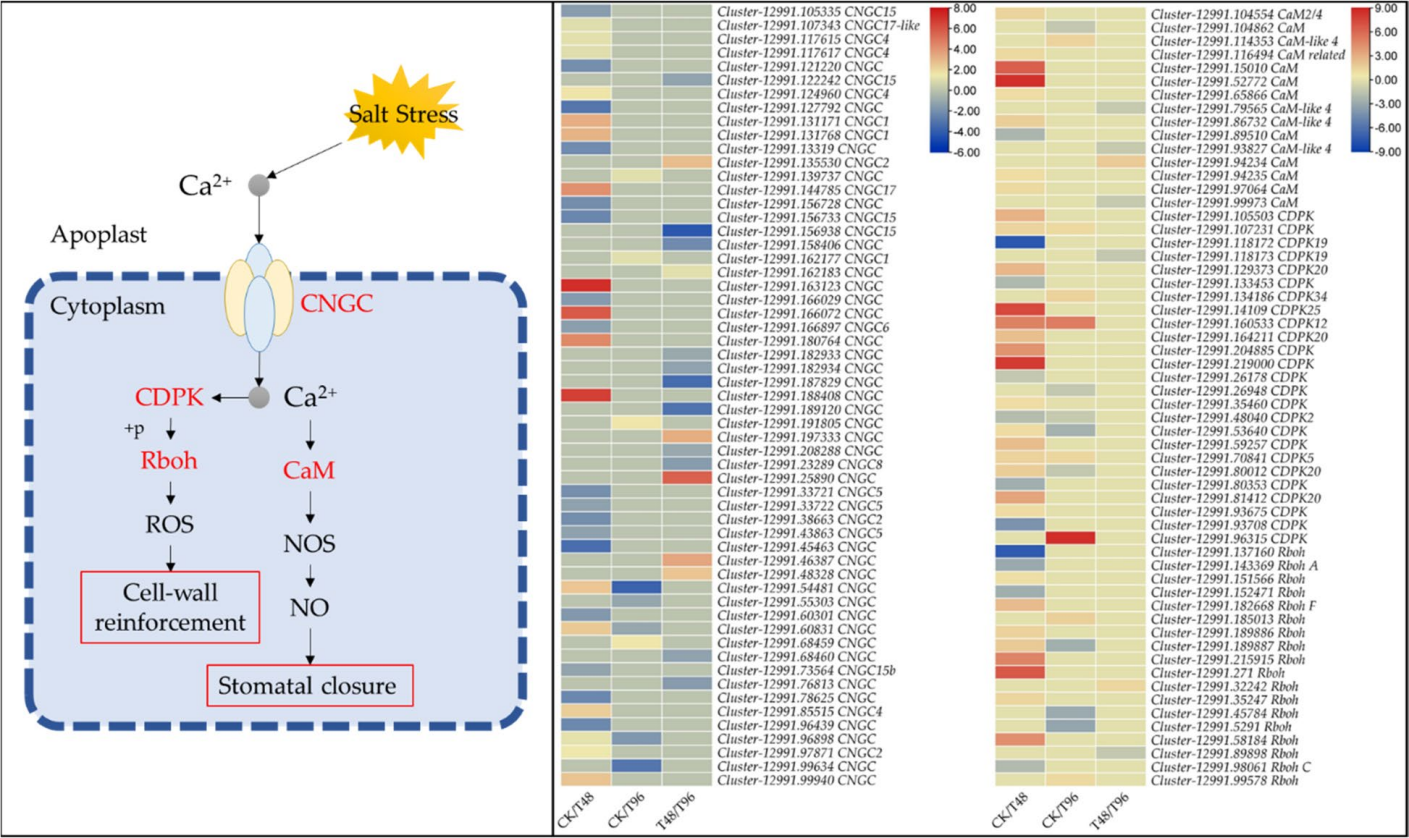
Changes in expressions in Ca.^2+^ signaling-related genes in *C. ensifolium* leaves under salt stress. The gene names with red color were differentially expressed between salt stress treatments and CK. The heatmaps represent log2 Foldchange values of the genes/transcripts. CNGC; cyclic nucleotide-gated channel, CDPK; calcium-dependent protein kinase, CaM; Calmodulin, Rboh; Respiratory burst oxidase, ROS; reactive oxygen species, NOS; Nitric-oxide synthase, NO; Nitric oxide. The red outline indicates the downstream changes (see supplementary Table 2 for cell wall and stomata-related gene expression changes)

Salt stress is perceived as an excess of Na^+^ that triggers an increase in cytosolic Ca^2+^ levels and downstream signaling cascades. In this regard, a large number of transcripts annotated as cyclic nucleotide-gated channels (CNGCs) were upregulated in T48 (17) and T96 (4) compared to CK. Most of the CNGCs that were co-expressed in both salt treatments were downregulated in T96 compared to T48. Once CNGCs transport Ca^2+^ from the apoplast to the cytosol, it is sensed by calcium-dependent protein kinases (CDPKs) and calmodulins (CaMs). We observed the upregulation of 15 of 21 CDPKs, 8 of 9 CaMs, and eight respiratory burst oxidases (Rboh) in T48 compared to CK whereas, a relatively smaller number of CDPKs (5 of 9), CaMs (1 of 2), and two Rbohs were upregulated in T96 compared to CK (Fig. 4). These changes in expressions suggest that Ca^2+^ is transported into the cytosol and downstream signals are sent for the generation of ROS and nitric oxide (NO) to induce cell-wall remodeling and stomal closure, respectively. These propositions are based on the observed expression changes and known roles of CNGCs, CDPKs, CaMs, and Rboh [25], which remain to be confirmed by targeted studies in this species. Nevertheless, the relatively higher number of DEGs associated with Ca^2+^ transport and signal transduction in T48 compared to T96 suggests that salt stress had caused damage by 48 h.

### Expression changes in stomata closure and cell-wall-related genes

Since salt stress triggers the formation of ROS and NO in response to changes in Ca^2+^ levels and downstream signals cause cell wall reinforcement and stomata closure [26], we searched the genes against the keywords “stomata” and “cell-wall” and found 154 and 921 transcripts, respectively. Major genes including CO_2_-response secreted protease, hexokinase, dihydroceramindase, cytochrome 1 and 2, Rboh, sodium/hydrogen exchanger 8 (NHX8), phototropin, MAPK 17/18, heme oxygenase, mitochondrial pyruvate carrier-1, and nitrate transporter were upregulated in response to salt stress. In general, the expression of genes related to stomata and cell-wall was higher in T48 followed by T96 and CK, which is consistent with the physiological data and the expression of genes related to calcium signaling. The known functions of these genes and their expression trends in T48 and T96 suggest that *C. ensifolium* undergoes changes in stomata development [27] and guard cell related functions under salt stress [28, 29]. Moreover, genes/TFs related to stomata closure, e.g., SPEECHLESS, were downregulated in T48 compared to CK and in T96 compared to T48 [30, 31]. In addition, one of the five MYB61 transcripts showed higher expression in T48 compared to CK. Interestingly, most cell-wall related genes are expressed only in CKvsT48. In particular, the small subunit ribosomal proteins (S9e, S20e, S24e, S27e, S5e, S10e, S16e) large subunit ribosomal protein l7e, cellulose synthase A (CESA), catalase (CAT), V-type H^+^-transporting ATPase (V-ATPase), endoglucanases, beta-mannan synthase, expansins, auxin-binding proteins, POD, pectinesterases were upregulated in T48 compared to CK (Supplementary Table 2). Overall, the differential expression of stomata-related genes suggests that salt-stress may lead to reduced stomatal density [31] and increased stomata closure [30]. Furthermore, extensive rearrangements and remodeling may occur in the cell-wall [32].

### Expression changes in ROS-scavenging-related genes

**Fig. 5:**
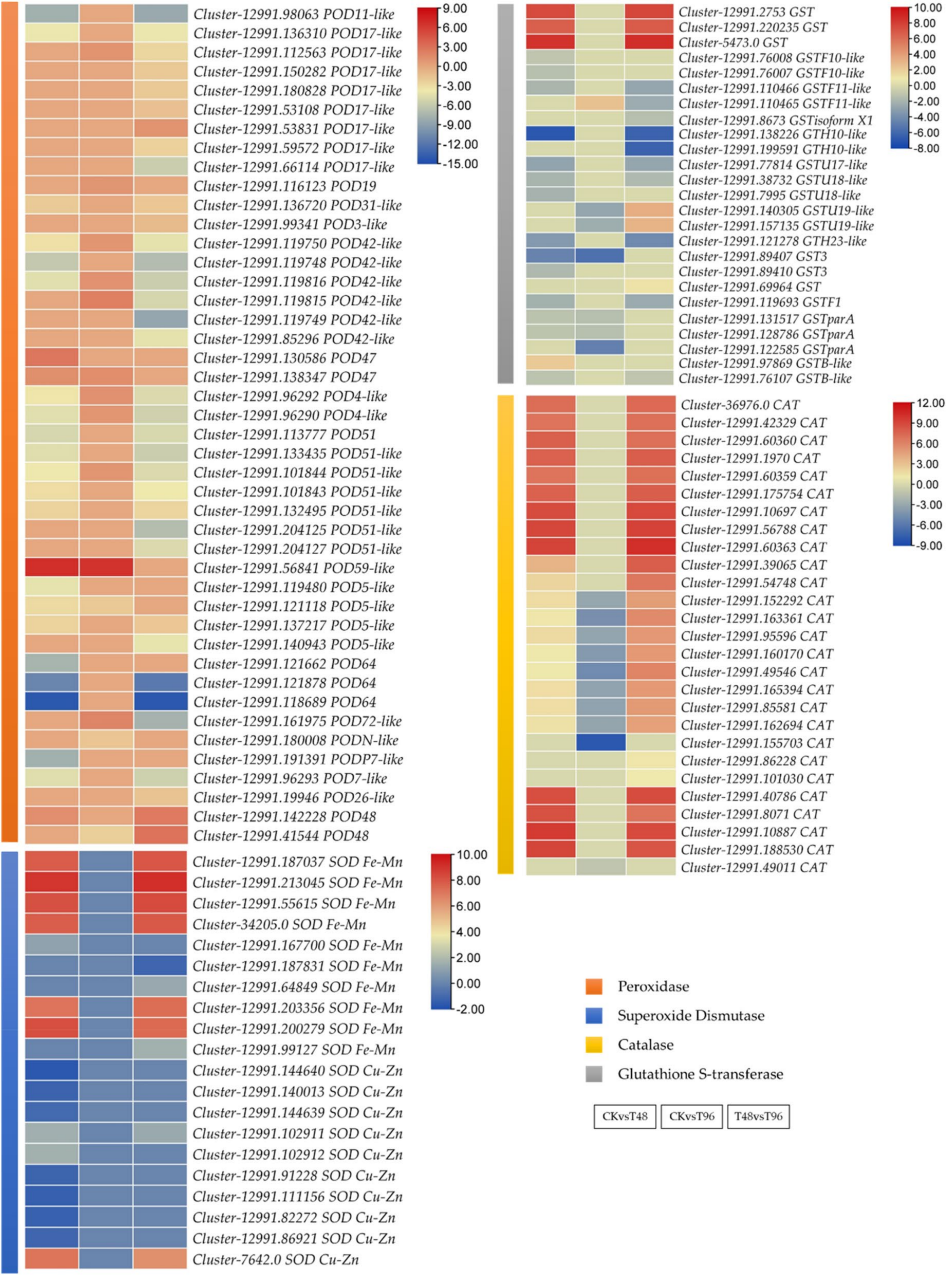
Differential expression of ROS-scavenging-related genes in *C. ensifolium* leaves under salt stress. The heatmaps represent log2 Foldchange values of the transcripts. The color bars represent different antioxidative genes

Comparative *C. ensifolium* leaf transcriptome data revealed differential expression of 116 transcripts annotated as SOD, CAT, GST, and POD. Four PODs (two POD47s, one POD59-like, and one POD48), 23 CATs, four GSTs, and 10 SODs (Fe–Mn family members) were upregulated in T48 compared to CK. On the other hand, the expression of a large number of PODs (21), GSTs (14), and SODs (7) was decreased in T48 compared to CK. Notably, only one SOD (*Cluster-12991.*99127) was expressed in T96. It was expressed only in response to salt stress i.e., in T48 (higher) and T96 (lower), suggesting that the activity of SOD may not change further after T48. This is consistent with the observation that the activity of SOD didn’t change significantly from T48 to T96 (Fig. 1). Most PODs and a GSTF11-like were upregulated in T96 compared to CK, whereas all CAT transcripts showed reduced expression in T96 compared to CK. Interestingly, the expression of CAT and SOD increased in T96 compared to T48, whereas POD and GSTs showed mixed expression patterns (Fig. 5). The changes in the upregulated POD, SOD, and GSTs are consistent with their respective activities (Fig. 1). These expression changes indicate that salt-stress activates antioxidant enzymes in *C. ensifolium* leaves.

### Expression changes in salt-stress response regulation related genes

#### Expression changes in ion-balance related genes

Plants have evolved mechanisms to maintain ionic balance (Na^+^ and H^+^) during salt-stress [33]. To understand how salt stress affects the expression of genes related to ion balance, we searched for SOS pathway genes. Two SOS2 and SOS5 transcripts were upregulated in T48 and T96 compared to CK. The SOS system regulated transporters (V-ATPases and H^+^-PPases) were upregulated in T48 and T96 compared to CK and downregulated in T96 compared to T48. However, compared to CKvsT48, only a small number of genes were differentially expressed in CKvsT96. Additionally, the regulators of SOS2, i.e. protein kinases (PKS), ABA-insensitive (ABI), and GIGANTEA (GI), were also differentially expressed in the salt treated leaves compared to CK. Furthermore, multiple ANNEXIN transcripts (ANND1-like, ANND3-like, ANND4, and ANND4-like) were also differentially expressed; these were mostly downregulated in T48 compared to CK and upregulated in T96 compared to T48. Apart from these, two glycogen synthase kinase 3 beta (GSK3β) were upregulated in T48 and T96 (one in each) compared to CK. GSK3 is known to interact with SOS2 and promote plant growth [34] (Supplementary Table 2).

In addition to maintaining a balance of Na^+^ and H^+^ under salt stress, plants must also modulate Na^+^/K^+^ homeostasis to maintain a high K^+^/Na^+^ ratio. In this regard, we observed the downregulation of potassium channel AKT1-like in T48 and T96 compared to CK, and upregulation in T96 compared to T48. An AKT2 was upregulated in T48 compared to CK. Additionally, the potassium channel KAT3 and KAT3-like were also differentially expressed between salt treatments and CK. The CBL-interacting protein kinases (CIPKs) that interact with AKT, such as were also differentially expressed; CIPK11-like, CIPK5, and CIPK5-like were downregulated in T48 compared to T96. Furthermore, a large number of PP2Cs, cation/calcium exchangers (CCXs), and vacuolar cation/calcium exchangers (CCAs) also showed variable expression patterns in the compared treatments and CK (Supplementary Table 2). These results indicate that *C. ensifolium* activates several mechanisms to maintain Na^+^ and H^+^ ion balance and Na^+^/K^+^ ratios under salt stress.

#### Expression changes in homeostasis related genes

Plant cells experiencing salt stress require osmotic homeostasis to prevent the reduction of turgor pressure, shrinkage of plasma membrane, and physical changes in the cells [33]. In this regard, protein kinases are functionally important. We observed that MAPKs (MAPK3, 6, and 8) were upregulated in T48 and T96 compared to CK. Whereas, one MAPKK2 and one MAPKK9 were upregulated and downregulated, respectively, in salt treatments compared to CK. Furthermore, the MAPKKK1s were upregulated, MAPKKK17/18 were downregulated, and MAPKKK (ANP1s) and MAPKKK (YODAs) were variedly expressed in salt-treated leaves compared to the CK (Supplementary Table 2). These changes indicate that *C. ensifolium* leaves initiate a MAPK signaling cascade to mitigate the effects of salt stress.

#### Changes in phytohormone (ABA and BR) signaling

Considering the essential role of ABA and BR signaling in salt-stress responses [25, 33], we searched for related differentially expressed transcripts. The ABA receptor PYR/PYL and PP2C transcripts were upregulated and downregulated, respectively, in salt stressed leaves indicating an increased perception of ABA. Whereas the serine/threonine-protein kinase SRK2s and ABA responsive element binding factors (ABFs) were upregulated in T48 compared to CK and T96 compared to T48. Other than ABA signaling genes, A large number of brassinosteroid insensitive 1-associated receptor kinase 1 s (BAK1s), protein brassinosteroid insensitive 1 s (BRI1s), and xyloglucan:xyloglucosyl transferases TCH4 showed variable expression patterns in salt stressed leaves compared to CK; A relatively higher number of BAK1, BRI1, and TCH4 transcripts showed reduced expression in salt stressed leaves. However, the brassinosteroid resistant (BZR) transcripts were only downregulated in T96 compared to T48. In contrast, the BRI1 kinase inhibitor 1 (BKI1) transcripts were upregulated in T48 compared to CK and T96 compared to T48 (Supplementary Table 2). These changes in expression suggest that salt-stress induced ABA and BR signaling in *C. ensifolium* leaves may affect stomatal closure and cell division, respectively [25, 33].

#### Expression changes in gene enriched in photosynthesis and related pathways

Finally, we searched for expression changes in genes enriched in photosynthesis and related pathways i.e., antenna proteins and carbon fixation in photosynthetic organisms. The expression of most of the photosystem (PS) proteins i.e., PS1 (PSI subunits II, III, IV, V, VI, IX, X, PsaN, and PsaO) and PSII (10 kDa, 13 kDa, 22 kDa, CP47 chlorophyll protein, and oxygen-evolving enhancer protein 2) were increased in T48 compared to CK. Notably, relatively minor expression variations were observed for the PSII genes compared to PSI genes. When salt stress was prolonged to 96 h, the expressions of the PSI and PSII genes were decreased compared to CK and T48 (Supplementary Table 2). The genes enriched in carbon fixation in photosynthetic organisms also showed similar expression patterns as of PSI genes. The light-harvesting complex I chlorophyll a/b binding protein 1 (Lhca1), Lhca3, Lhca4, and Lhca5 were upregulated in T48 and downregulated in T96 compared to CK. Whereas Lhca2 and Lhcb were mostly downregulated in both salt treatments compared to CK. These observations indicate that salt stress induces changes in the light-harvesting complex, photosynthesis, and carbon fixation. The longer the plant is under salt stress, the more damage it sustains.

#### Expression changes in transcription factors

Considering the importance of TFs in abiotic stress responses, we searched for expression changes in TFs (and transcriptional regulators, TRs) in the studied treatment comparisons. We found 825, 291, and 869 DEGs annotated as TFs/TRs in CKvsT48, CKvsT96, and T48vsT96, respectively. These TFs/TRs belonged to 77 families. The most differentially expressed TFs in CKvsT48 were bHLH (45), followed by MYB (43), NAC (38), MYB-related (35), and C3H (30). Highly upregulated TFs in T48 compared to CK were B3-ARF, SET, bHLH, CSD, and GARP-G2-like. Whereas, the most downregulated TFs in T48 compared to CK were B3-ARF, bHLH, GRAS, MADS-M-type, and HB-other. The most upregulated TFs in T96 compared to CK were classified as SET, HB-HD-ZIP, MYB-related, GARP-G2-like, and PLATZ. Whereas the downregulated TFs in T96 compared to CK were bHLH, HSF, MADS-MIKC, SNF2, and B3-ARF. These results indicate that a relatively higher number of TFs/TRs are activated in response to salt stress after 48 h compared to 96 h. Fifteen transcripts classified as HSF, SET, MYB-related, WRKY, NAC, LOB, NF-YC, Mterf, and bHLH families, showed a decrease in expression with increasing duration of salt stress. In contrast, 19 TFs/TRs showed increasing expression trends with the increase in salt stress time. These included members of the AP2/ERF-ERF, TRAF, SBP, C3H, PHD, MYB-related, SNF2, mTERF, bHLH, FAR1, Tify, and HB-HD-ZIP TF/TR families (Supplementary Table 3).

#### Salt tolerance analysis of transgenic Arabidopsis

**Fig. 6:**
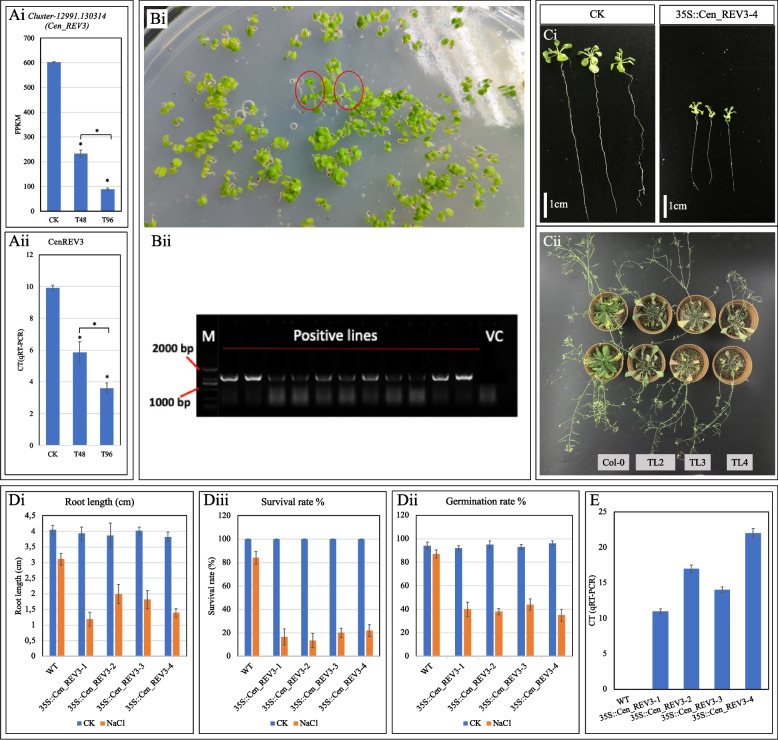
Salt stress tolerance analyses of the *CenREV3 Arabidopsis* overexpression lines. Ai) Expression (FPKM) and Aii) CT (qRT-PCR) of *CenREV3* in *C. ensifolium* leaves at CK, T48, and T96. The values are the mean ± SD of five replicates (*p* < 0.05). The asterisk sign (*) represents significant differences. B) Selection and confirmation of transgenic *Arabidopsis* lines. Bi) Transgenic lines selected on selection medium. Bii) Confirmation of positive transgenic *Arabidopsis* lines by PCR, where M = ladder, 2–11 wells are PCR confirmation results, and the last well represents vector control. Ci) Comparison of wild type and transgenic *Arabidopsis* seedlings under salt stress conditions (100 mM NaCl) for seven days. Cii) Morphological comparison of transgenic lines (TL) and wild-type after salt stress treatment (100 mM NaCl). Two rows present two different plants for the same line. C) Morphological analysis of the *CenREV3* overexpression lines; Di) root length, Dii) germination %, and Diii) Survival %. E) Expression (CT (qRT-PCR) of *CenREV3* in four transgenic lines. The qRT-PCR was repeated five times and the means were presented. Original pictures are presented in Supplementary data 1

Comparative transcriptome analysis of salt stress and CK *C. ensifolium* revealed several salt-stress-responsive genes/TFs for which our laboratory started in-depth functional investigations. As of now, we have selected 100 genes/TFs (data not shown) from the DEGs for functional characterization because of their interesting expression profiles and stress-responsive GO annotations. Here, we present the functional characterization of *CenREV3* in *Arabidopsis thaliana*. The FPKM values of *Cluster-12991.130314* (*CenREV3*) decreased significantly in T48 and T96 successively, indicating that with the increase in salt stress duration, its expression decreases compared to CK (Fig. 6Ai). The relative expression of *CenREV3* measured by qRT-PCR confirmed the FPKM expression trend (Fig. 6Aii). These FPKM and qRT-PCR data, as well as a similar expression trend in another proteome-based investigation in our laboratory (data not shown), suggested that it is a potential regulator of salt-stress tolerance in *C. ensifolium*. It was annotated for several GO terms e.g., DNA binding, TF activity, response to ABA, GA, JA, SA, response to salt stress, and transcription. Therefore, we hypothesized that *CenREV3* is a negative regulator of salt stress tolerance in *C. ensifolium.* Its sequence analysis showed that it is a 1,233 bp long gene, annotated as protein REVEILLE 3, and contains a Myb-like DNA-binding domain, thus we named it *CenREV3* (Supplementary Table 3).

To further examine its function, we successfully obtained 11 Arabidopsis transgenic homozygous T3 lines (TLs) expressing *CenREV3* by the floral dip method. Four of these homozygous TLs were selected for further analysis. Positive transformants were selected and confirmed by PCR (Fig. 6B; Supplementary data 1). The selected *Arabidopsis* TLs were tested for salt tolerance with 0 and 100 mM NaCl. The morphological measurements showed that there were no significant differences between TLs and WT for root length (cm), germination rate (%), and survival rate (%) under normal conditions (Fig. 6C; Supplementary data 1). However, the root length, germination rate, and survival rate were significantly reduced in TLs compared to WT under 100 mM NaCl stress (Fig. 6D; Supplementary data 1). The expression analysis indicated that *CenREV3* was highly expressed in the four TLs (Fig. 6E; Supplementary data 1). These results establish that a reduction in the expression of *CenREV3* is related to salt stress tolerance.

## Discussion

Considering the high ornamental/floristic value and decline of natural *C. ensifolium* populations*,* and increasing soil salinity in China [8, 35], we investigated the leaf responses to salt stress by transcriptome sequencing. When plants are under salt stress, the solution in the root system has a low water potential and therefore cannot supply water to the leaves, resulting in an imbalance in ion concentrations [36]. In such situations, plant leaves suffer both nutrient and ion losses [37]. Therefore, we selected leaves for physiological analyses and subsequent transcriptome sequencing. Since leaves are not in direct contact with the salt solution in soil but roots are, leaves sense salt stress after the leaf water potential is reduced, therefore, we opted to study the leaf responses to salt stress after 48 and 96 h. Considering the limited/no availability of data sets on *C. ensifolium’s* salt stress responses, our study provides a preliminary but comprehensive insight into this issue. These results will serve as a reference for future investigations toward better resolution at extended time points.

### C. ensifolium leaves exhibit expression changes in salt stress sensing related genes

Salt stress sensing initiates complex signaling cascades. Increased Na^+^ content, as observed in this study, is one of the earliest responses to salt-stress that triggers alternations in K^+^ and Ca^2+^ levels. This change in Na^+^ content in *C. ensifolium* leaves is consistent with the earlier reports on dune spinach [38] and cucumber [39]. The increased Na^+^ content can trigger changes (increase) in Ca^2+^ levels by inducing ionic and osmotic stress [33], which can be achieved through the activation of Ca^2+^ channels (CNGC) [14]. The differential expression of CNGCs, CDPKs, CaMs, and Rboh indicates elevated Ca^2+^ levels in *C. ensifolium* leaves in response to salt stress, thus preparing the leaves for stomata closure and changes in the cell wall [40]. The CNGCs and CaMs interact by forming a molecular switch to operate calcium channels based on Ca^2+^ levels [41]. Additionally, CNGCs can also provide a pathway through which Ca^2+^ is transported across the plasma membrane and facilitate cytosolic Ca^2+^ elevation [42]. Thus, the higher expression of CNGCs in T48 than in T96 (Fig. 4) suggests that Ca^2+^ levels was higher at the first time point and increased in the second time point. The physiological data suggested that leaves were more damaged in T48 than in T96 (Fig. 1), further supporting the possible role of CNGCs as stated above. The known role and higher expressions of CO_2_-response secreted protease [27], hexokinase [43], Rboh [44], and NHX8 in salt-stressed *C. ensifolium* suggest the involvement of several genes in the regulation of stomatal closure. Similarly, the differential expression of 921 cell wall related genes belonging to different gene families (Supplementary Table 2) signifies that salt stress induces structural modifications in the cell wall, and prolongation of the stress may compromise the cell wall integrity [40]. This assumption is based on the observed expression patterns and known functions of CESAs, endoglucanases, beta-mannan synthases, pectinesterases, and other cell wall related genes [45].

**Fig. 7:**
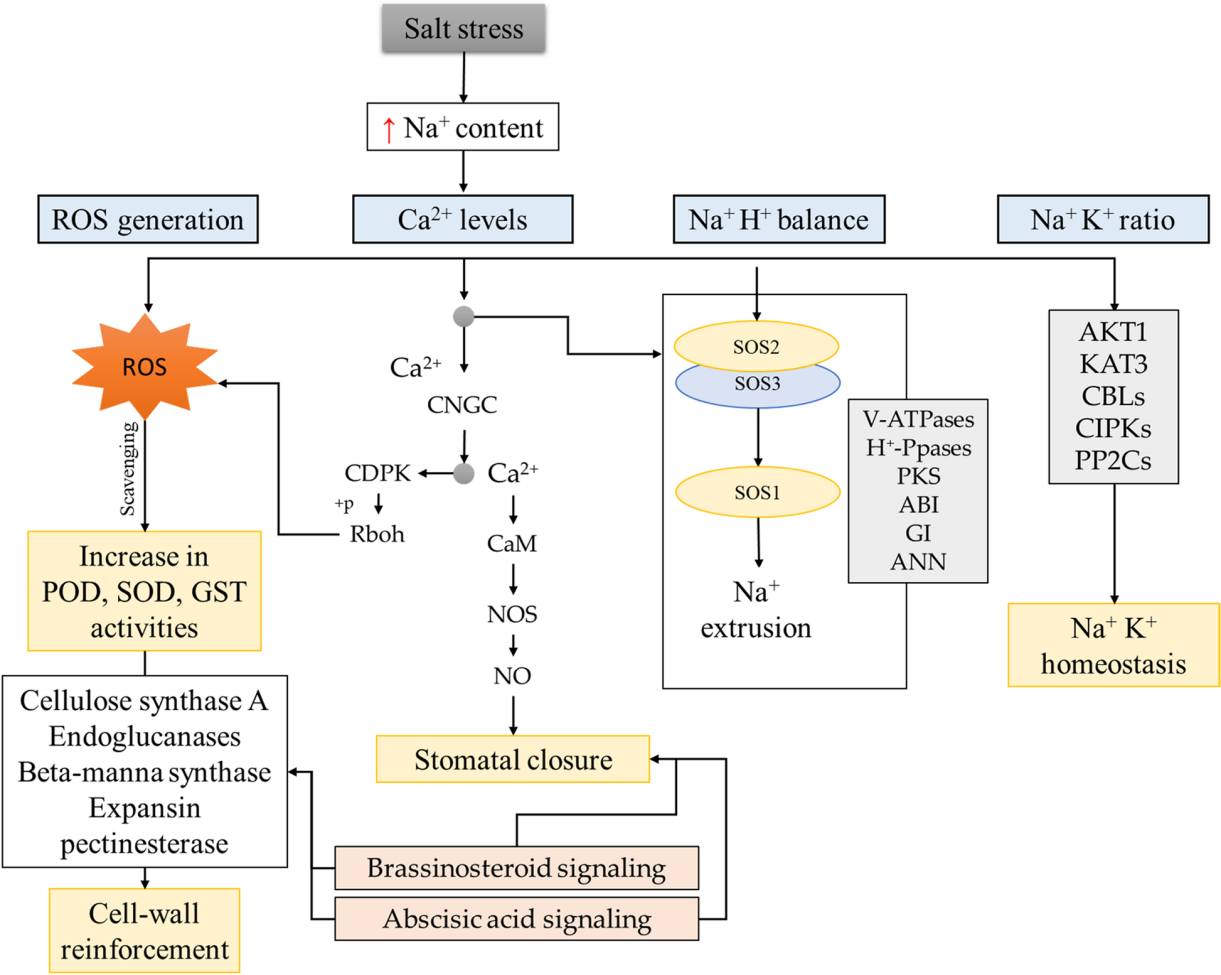
Salt stress responses in *C. ensifolium* leaf. Salt stress results in changes in Na^+^ content, which affects Ca^2+^ levels. After salt stress is sensed, the ROS are generated, the Na^+^ H^+^ balance is disturbed, and Na^+^/K^+^ ratios are changed. The increased ROS are scavenged by antioxidant enzymes. The higher Ca^2+^ levels initiate a cascade of signaling that results in the expression of genes that govern cell-wall reinforcement, stomatal closure, and the SOS pathway. Several genes are activated for the homeostasis of Na^+^ and K^+^ ions. Brassinosteroid and abscisic acid signaling also takes part in salt stress responses

Upon sensing salt stress, plants experience oxidative stress, which is evident from the overaccumulation of ROS. To cope with the excess ROS, plants utilize endogenous enzymatic and non-enzymatic antioxidant defense mechanisms. The increased expressions of POD (POD47, POD59-like, and POD48), SOD, and GST (*Cluster-12991.2753, Cluster-12991.220235, Cluster-5473.0,* and *Cluster-12991.97869*) in salt stressed *C. ensifolium* (Fig. 1) in T48 compared to CK and decreased expressions in T96 compared to T48 are consistent with the physiological data (Fig. 1). These results showcase the ability of *C. ensifolium* to initiate an enzymatic antioxidant defense mechanism against salt stress. The downregulation of many PODs in T48 compared to CK (Fig. 5) in contrast to POD enzyme activity in T48 is interesting (Fig. 1). Similar findings have been reported in other plants e.g., in wheat, where different POD genes showed variable expressions (upregulation and downregulation) under abiotic stress [46]. A possible explanation for these results could be the fact that except for ROS scavenging, PODs are also involved in a variety of metabolic functions in plants such as cell wall cross linking, lignification, auxin catabolism, and secondary metabolism [47]. Furthermore, their expression is complicated because they can be expressed in different tissues at different times under the influence of different stresses, so information about the timing and tissues is important for comparing the results with other species. Therefore, the downregulation of multiple PODs can be regarded as preliminary data for future studies on abiotic stress in *C. ensifolium*. Similarly, the downregulation of some GSTs may be associated with cell division and leaf water content under abiotic stress [48]. Since our objective in this study is to provide a global transcriptome profile in response to salt stress, therefore, these proposed functions should be explored in specific gene characterization studies. Nevertheless, the POD, SO, and GST expression changes in T48 and T96 (Fig. 5; Supplementary Table 2) indicate that similar to *Catharanthus roseus* [49] and other orchids [50], *C. ensifolium* activates specific genes to ameliorate salt stress (Fig. 7).

### Salt stress initiates expression changes in ion balance and homeostasis related genes

The salt stress results in the accumulation of Na^+^ to toxic levels leading to disruptions in ion homeostasis [51]. Plants have evolved sophisticated systems to maintain cytosolic Na^+^ levels such as exchanging Na^+^ with H^+^ through NHXs present in the plasma membrane [52]. This exchange is modulated by the SOS pathway, which itself is triggered by cytosolic Ca^2+^ [26]. The upregulation of SOS2 and SOS5 transcripts in response to salt stress indicates that *C. ensifolium,* similar to other plants, recruits SOS pathway members for ion balancing. The Ca^2+^ signal perception activates SOS2, which then interacts with other SOS members to enhance salt stress tolerance. The upregulation of V-ATPases and H^+^-PPases in T48 compared to CK and downregulation in T96 compared to T48 indicate that increased exposure to salt stress may result in decreased SOS-regulation of salt stress [53]. This is further supported by the differential expression of a relatively smaller number of genes related to ion homeostasis in CKvsT96 compared to CKvsT48 (Supplementary Table 2). Furthermore, changes in the expression of PKS, ABI, and GI transcripts in T48 and T96 compared to CK indicate their role in the regulation of SOS2 expression to maintain ion balance under the influence of salt stress [54–56]. Additionally, the differential expression of ANNs also confirms the above statement as they can fine-tune Ca^2+^ signaling by interacting with the SOS2 [57, 58]. The higher expression of ANNs in T96 than in T48 indicates that *C. ensifolium* tries to overcome the prolonged salt stress by activating these genes as they are involved in salt-stress response alleviation [57, 58]. Since they are also regulated in a light-dependent manner [57], their expressions cannot be specifically explained because we did not include light as a variable in this experiment. Nevertheless, the expressions of ANNs and phytohormone-related genes’ indicate their possible interaction [59]. Particularly, the upregulation of GSK3β in both salt treatments suggests that the SOS pathway also interacts with BR signaling to elicit salt stress responses [34]. The changes in photosynthesis – antenna proteins, photosynthesis, and carbon fixation in photosynthetic organisms are also relevant to expression changes discussed above [60] (Fig. 7).

In addition to Na^+^/H^+^ exchange, plants must also maintain the K^+^/Na^+^ ratio under salinity stress. In this regard, the role of K^+^ transporters i.e., AKT1 and KAT3, is important as they mediate K^+^ absorption, release, and transport. Salt stress inhibits AKT1 activity through the action of CBLs [18, 61]. The reduced expression of AKT1 and differential expressions of CIPKs and PP2Cs in T48 and T96 confirm that salt stress induces K^+^ concentration changes in *C. ensifolium* [18]. These observations are consistent with the reports that CIPKs (CIPK23) bind with AKT1 to enhance its activity during salt stress [62], whereas the PP2 negatively regulates the CIPK23-activated AKT1 by dephosphorylating the AKT1 [63]. Similarly, the differential expressions of CCXs and CCAs indicate that *C. ensifolium* tries to maintain Na^+^ concentrations across the plasma membrane by activating these genes [64]. Taken together, the results presented in this study indicate that this plant species recruits multiple genes for Ca^2+^ signaling, Na^+^ extrusion, Na^+^/H^+^ exchange, and maintenance of Na^+^/K^+^ ratios to cope with salt stress (Fig. 7).

Salt stressed plants also experience osmotic stress, which causes biophysical changes in cells. The increased MDA content in T48 and T96 compared to CK (Fig. 1) indicates that salt stress induced oxidative stress increases lipid peroxidation in *C. ensifolium* leaves [65]. To tolerate these changes, osmotic signaling pathways accumulate osmolytes, which change osmotic potential in cytosolic compartments and act as signals to induce ABA [66]. Since protein kinases act as junction points for swift osmoregulation and signaling under salt stress scenarios, therefore, the expression changes in MAPKs, MAPKKs, and MAPKKKs indicate that *C. ensifolium* leaves experience osmolyte biosynthesis changes (Supplementary Table 2) [67]. Furthermore, MAPKs also confer salt stress tolerance and induce defense responses in plants. For example, MPK5, MPK3, and MAPK6 improved salt stress tolerance in tobacco [68] and rice [69]. Therefore, sequencing these expression changes in *C. ensifolium* leaves suggests osmotic homeostasis related signal transduction is activated during salt stress.

### Salt stress induces abscisic acid and brassinosteroid signaling

Salt stress leads to an increase in ABA biosynthesis and signaling, resulting in changes in the expression of downstream genes [70]. The upregulation of the ABA receptors PYR/PYLs in T48 and T96 indicates that ABA biosynthesis is increased [71], which in turn inhibits PP2Cs as evident from their downregulation in salt stressed leaves [72]. The upregulation of the SRK2s and ABFs in T48 and T96 further processes the ABA signals downstream to produce an effect [73, 74] because the absence/suppression of PP2Cs activates SnRKs. As discussed above (3.1), genes related to stomata closure were also differentially expressed between CK and salt treatments, therefore, our results imply that ABA has a role in salt stress responses in *C. ensifolium.* Additionally, BRs are known to mediate physiological processes under salt stress [75]. The differential regulation of BAK1, BRI1, BZR, BKI1, and TCH4 genes in the studied treatment comparisons indicate that BR-signaling is activated in *C. ensifolium* under salt stress. Notably, the concomitant expression changes in BAK1 and PYR/PYLs indicate the possible interplay of ABA and BRs in *C. ensifolium* under salt stress as previously described in *Arabidopsis* [76]. Moreover, the BRI1 and BAK1 expression changes suggest that the BRI1-BAK1 heterodimer may also be involved in initiating a phosphorylation relay cascade in *C. ensifolium* under salt stress [77]. These results indicate that *C. ensifolium* leaves activate ABA and BR signaling cascades to induce changes in downstream processes such as stomata closure and cell wall modification.

### Salt stress modulates large scale expression changes in transcription factors in C. ensifolium leaves

Transcription factors play integral roles in regulating abiotic stress responses in plants. A large number of TFs from bZIP, AP2/ERF, WRKY, NAC, bHLH, and MYB families have been implicated in salt stress responses in *Arabidopsis* [78]. The results that 825 TFs belonging to more than 70 families were differentially expressed in CKvsT48 indicate their importance in the response to salt stress in *C. ensifolium* (Supplementary Table 3)*.* The result that TF B3-ARF was strongly upregulated in T48 is consistent with the earlier reports that most ARFs in *Solanum tuberosum* were responsive to salt stress [79]. Similarly, the increased expressions of bHLH, SET, CSD, and GARP-G2-like TFs/TRs indicate *C. ensifolium* undergoes large-scale transcriptional changes under salt stress. The highest number of differentially expressed bHLH TF family members is an important observation because these TFs are involved in modulating resilience to salt stress in sorghum [80], *Arabidopsis* [81], and sugar beet [82]. Similarly, the large number of differential TFs classified as MYB and NAC implies that similar to other plants [83, 84], *C. ensifolium* recruits them to modulate transcriptional changes under salt stress. Likewise, the expression changes in WRKY TFs suggest their role in salt stress regulation in *C. ensifolium.* WRKY is one of the largest TF families in plants and its different members have been implicated in salt stress tolerance mechanisms in different crop plants [85–87]. Taken together, the results indicate that a large number of TF families are involved in salt stress responses in *C. ensifolium* and provide novel expression patterns and candidate TFs for future characterization studies.

### CenREN3 is a negative regulator of salt stress tolerance

The REVEILLE genes from different plant species have been characterized for their role in different traits. In Arabidopsis, they are known to regulate the circadian clock rhythm by controlling cell size (for example *REVEILLE 8*) [21], cold tolerance, circadian-regulated processes, and seed germination (*AtREV2*) [22, 23]. The presence of a Myb-like DNA binding domain in *CenREV3* suggests that it may regulate salt stress tolerance. Previously, MYB TFs have been associated with salt stress tolerance in many plant species. For example, the R2R3-type MYB gene *OsMYB91* was negatively associated with plant growth and salt stress tolerance in *Arabidopsis* [24]. The annotation results that *CenREV3* was linked to several GO terms including phytohormone responses and salt stress suggest that it could be a regulator of salt stress tolerance e.g., *AtMYB1* directly targets ABA biosynthesis and signal transduction under salt stress [88]. The increased expression of *CenREV3* in the four TLs and the corresponding decrease in morphological traits i.e., root length, germination rate %, and survival %, show that it is a negative regulator of salt stress tolerance. However, how *CenREV3* interacts with other signaling and salt stress responsive genes should be investigated by transcriptome sequencing and/or metabolome profiling of the *CenREV3* overexpressing tissues/plants. Nevertheless, these results highlight the role of *CenREV3* under the studied conditions and provide an early understanding of its role in plant survival under salt stress conditions. In addition to *CenREV3*, we are also characterizing 99 salt stress responsive genes and preparing a gene interaction map and gene function catalog in *C. ensifolium*. We believe that these resources will accelerate the improvement of *C. ensifolium* and related species in abiotic stress tolerance.

## Conclusion

In this study, we report that *C. ensifolium* initiates antioxidant defense responses such as increasing the activities to POD, SOD, and GST when challenged with 100 mM NaCl stress for 48 and 96 h. The MDA content increases significantly in salt stressed leaves. The increased salt stress was evident from the significant increase in Na^+^ levels. The comparative transcriptome sequencing of *C. ensifolium* leaves showed that genes related to salt sensing, ROS-scavenging, Ca^2+^ signaling, osmotic homeostasis, and phytohormone signaling (particularly ABA and BRs) are differentially expressed. *Cymbidium ensifolium* leaf undergoes changes in photosynthesis – antenna proteins, photosynthesis, and carbon fixation in photosynthesis pathways-related genes under the influence of salt stress (Fig. 7). The *CenREV3* genes’ expression decreases with the increase in salt stress duration. The *CenREV3* overexpressing transgenic *Arabidopsis* lines showed reduced root length, germination %, and survival % under salt stress, indicating that *CenREV3* is a negative regulator of salt stress tolerance.

## Material and methods

### Plant material and salt stress treatments

Six-month-old *Cymbidium ensifolium* var. ‘Shi zhang hong’ plants grown in an orchid germplasm nursery at Fujian Forestry Science and Technology Experimental Center, China, were used in this study. No prior permissions are required to work on this species. Voucher specimens are available in the herbarium of the Genebank of the Fujian Forestry Science and Technology Experimental Center under the number FY2510BT0A. The plant material was identified by Prof Yunpeng Luan (see author list). All relevant institutional, national, and international guidelines and laws were followed while conducting this experiment. The experimental plants were grown in plastic pots (20 × 20 cm^2^) and kept in a greenhouse. The air humidity, temperature, and shading were 80%, 25 ℃, and 80%, respectively. We selected uniform plants, divided them into five experimental groups, and applied zero (distilled water), 50, 100, 150, and 200 mM NaCl solution, respectively. The NaCl treatment was repeated every two days. After two rounds of NaCl solution treatment, the plants were allowed to recover for 14 days, and the mortality rate was determined. The 100 mM NaCl treatment was selected for further analyses because the mortality rate was 100% for 200 mM NaCl treatment. Triplicate leaf samples were collected after 0 (CK), 48 (T48), and 96 (T96) hours of 100 mM NaCl treatment and processed for RNA-sequencing.

### Physiological analyses

#### Antioxidant enzyme activity assays and lipid peroxidation analysis

*Cymbidium ensifolium* leaves (0.5 g) were ground in ice-cold 100 mM phosphate buffer (pH 7.5) and the homogenates were centrifuged at 1500 × *g* for 20 min at 4 ℃. The supernatants were then used to determine the activities of superoxide dismutase (SOD; EC 1.15.1.1) and peroxidase (POD; EC 1.11.1.7) by following a previously described method [89]. The glutathione S-transferase (GST; EC 2.5.1.18) activity and lipid peroxidation (malondialdehyde (MDA) content) was determined by the method reported by Peragón and Amores-Escobar [90] and Attri*, *et al*.* [91], respectively. The activity of POD was expressed as U·g fresh weight (FW) − 1, and SOD activity and MDA content were expressed as U·min^−1^·g FW^−1^ and nmol·g FW^−1^, respectively.

#### Na+ content determination

The Na^+^ content was determined as described previously [92]. Briefly, the samples were weighed on Teflon plates, transferred into DAP-60S pressure mineralization tubes, and added 2 mL 67% HNO_3_ and 3 mL H_2_O_2_. The tube contents were mixed and kept for 90 min at room temperature followed by placing them on a rotor of Berghof MWS-3 + Speedwave microwave (Berghof, Germany) for 60 min for mineralization at 100–190 ℃. The mineralizates were heated at 150 ℃ for evaporation. To the remaining moist residue, 1.5% HNO_3_ was added. This mixture was quantitatively transferred to tubes and added with demineralized water to obtain a final volume of 25 mL. The Na^+^ content was then determined using the AAS method on a Varian SpectrAA 100 (Varian, Mulgarve, Victoria, Australia) with SIPS at a wavelength of 589.0 nm.

#### Statistical analysis of the physiological and biochemical changes

The results between the salt treatment (48 and 96 h) and CK (0 h) were analyzed by comparing *F* values obtained from a one-way analysis of variance test (*p* < 0.05) using SPSS v. 13.0 package (Available online: http://spss.en.softonic.com/). The least significant differences (Post Hoc) test was used to compare means (*n* = 3).

### Transcriptome sequencing

#### RNA extraction, libraries preparation, and sequencing

Triplicate leaf samples stored at -80 ℃ were processed for RNA extraction and sequencing at Benagen Company Ltd., Wuhan, China (www.benagen.com). TRIzol reagent kit was used for the extraction of total RNAs. RNAs were enriched with oligodTs and interrupted randomly by adding the fragmentation buffer. The first strand cDNAs were synthesized and used for second strand synthesis. The double-stranded cDNA was purified using AMPure XP beads, end-repaired, A-tailed, ligated with sequencing adapters, and fragmented with AMPure XP beads. The final cDNA libraries were then obtained using size selection and PCR enrichment reactions. Libraries were then pooled and sequenced on Novaseq 6000 platform (Illumina, San Diego, CA, USA).

### Sequencing data analyses

The sequencing data were then processed and the sequencing error rate and GC content distribution were determined following a quality check of the raw data. Reads with adaptors, paired reads, and low-quality reads were removed. Clean readers were then used for BLAST [93] to compare the unigene sequences with different annotation databases including Nr [94], Pfam [95], Swiss-Prot [96], KEGG [97], and GO [98]. A reference sequence was first generated from the transcript sequences obtained from Trinity splicing. The clean reads for each replicate of treatment and CK group were analyzed by RSEM software [99]. The FPKM (expected number of Fragments Per Kilobase of transcript sequence per Million base pairs sequenced) values were determined using the featureCounts software [100]. Principal Component Analysis and PCC were computed in R. DESeq2 was used to determine the differential gene expression between salt treated groups and CK [101]. The DEGs were then enriched on the KEGG pathways [102]. Finally, we used the iTAK software to predict the transcription factors (TFs) [103].

### Vector construction and transgenics development

Based on the transcriptome analyses and another proteome-based study with similar conditions (data not shown), we selected a highly downregulated gene *Cluster-12991.130314 (CenREV3)* in T48 and T98 compared to CK. The gene was overexpressed in *Arabidopsis* using the vector ProkII under the control of the CaMV 35S. The cDNA was amplified using a forward primer (5’- CTGCCTGAGCCAGGCTATAC-3’) and a reverse primer (5’- GCGCAGGCATCATACAGAGA-3’). The PCR product was confirmed by sequencing. The gene fragment was digested with XbaI and KpnI, and cloned in a plant binary vector (ProkII). The promoter and terminator were CaMV 35S and NOS-T, respectively; 35S::CenREV3. The construct was introduced into *Agrobacterium tumefaciens* strain LBA4404 after sequencing and transformed into *Arabidopsis* (Col-0) by the floral dip method. Eleven transgenic homozygous T3 plants were obtained (selected based on a progeny test), of which, four were selected for downstream investigations. The expression of the transgene was checked using the qRT-PCR approach as described by Dossa et al. [92] with the primers (*ATACTIN2*forward: 5’-ACCTTGCTGGACGTGACCTTACTGAT-3’reverse: 5’-GTTGTCTCGTGGATTCCAGCAGC TT-3’; Cen_REV3forward: 5’-CTGCCTGAGCCAGGCTATAC-3’reverse: 5’-GCGCAGGCATCATACAGAGA-3’). Five replicate plants were used for all experiments, whereas the whole experiment was repeated twice at different periods.

### Salt stress treatment and agro-morphological analyses

Fifty seeds of wild type and the overexpressing Arabidopsis lines were sown on a half-strength Murashige and Skoog (MS) medium supplemented with 100 mM NaCl and kept at 4 ℃ for three days followed by shifting to a growth chamber. The temperature, light intensity, and relative humidity were 22 ℃, 120—150 μmol/m^2^.s, and 50%, respectively. The percentage of germinated seeds with green cotyledons was recorded after seven days. Next, the seeds of the wild type and the four T3 lines (*n* = 50 for each line) were plated on MS medium. Plates were placed vertically and kept for 10 days. The seedlings were then moved to a new MS medium supplemented with 100 mM NaCl. The root length was recorded (using a ruler) after one week. Finally, the *Arabidopsis* plants (wild type and the overexpressing lines, *n* = 100) were grown in soil for 25 days and irrigated biweekly with 100 mM NaCl solution for two weeks. The CK seedlings were devoid of NaCl treatment. The plants were then allowed to recover and the survival rate was recorded after three weeks. All the experiments were conducted in three replicates. Germination rate (%), root length (cm), and survival rate (%) were determined as reported earlier [104].

## Supplementary Information

**Additional file 1: SupplementaryFigure 1.**Annotation statistics of the DEGs in *C. ensifolium *leaves in differentdatabases. 

**Additional file 2: SupplementaryTable 1. **Statistics of C. ensifolium leaf sequencingchallenged with 100 mM NaCl stress for 48 and 96 hours.** SupplementaryTable 2. **Pathway specific differentially expressed genes inC. ensifolium leaf challenged with 100 mM NaCl stress for 48 and 96 hours.** SupplementaryTable 3. **Differentially expressed transcription factors inC. ensifolium leaf challenged with 100 mM NaCl stress for 48 and 96 hours.

**Additional file 3: Supplementarydata 1. **Original pictures used to prepare the Figure 6

## Data Availability

The raw RNA-seq data have been submitted to NCBI SRA under the project number: PRJNA790719. Voucher specimens are available in the herbarium of the Genebank of the Fujian Forestry Science and Technology Experimental Center under the number: FY2510BT0A.
