# Supplementary material for: Transcriptome analysis reveals the key pathways and candidate genes involved in salt stress responses in Cymbidium ensifolium leaves
Source: BMC Plant Biol. 2023 Feb 1;23:64. doi: 10.1186/s12870-023-04050-z (PMC9890885; doi:10.1186/s12870-023-04050-z)
Supplement: Supplementary file 2 — Additional file 2: SupplementaryTable 1. Statistics of C. ensifolium leaf sequencingchallenged with 100 mM NaCl stress for 48 and 96 hours. SupplementaryTable 2. Pathway specific differentially expressed genes inC. ensifolium leaf challenged with 100 mM NaCl stress for 48 and 96 hours. SupplementaryTable 3. Differentially expressed transcription factors inC. ensifolium leaf challenged with 100 mM NaCl stress for 48 and 96 hours. [file 12870_2023_4050_MOESM2_ESM.pptx]

## Slide 1
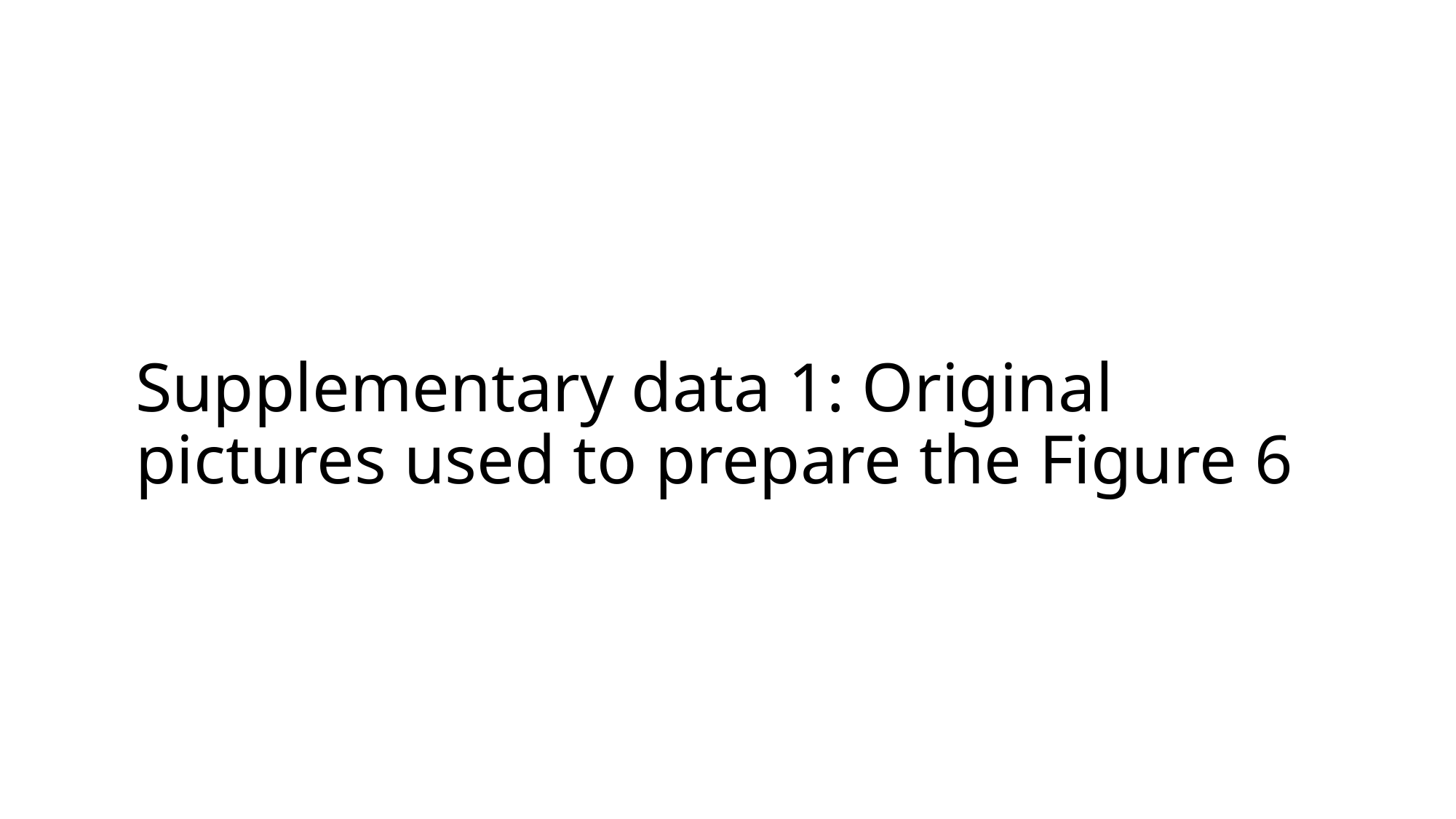

# Supplementary data 1: Original pictures used to prepare the Figure 6

## Slide 2
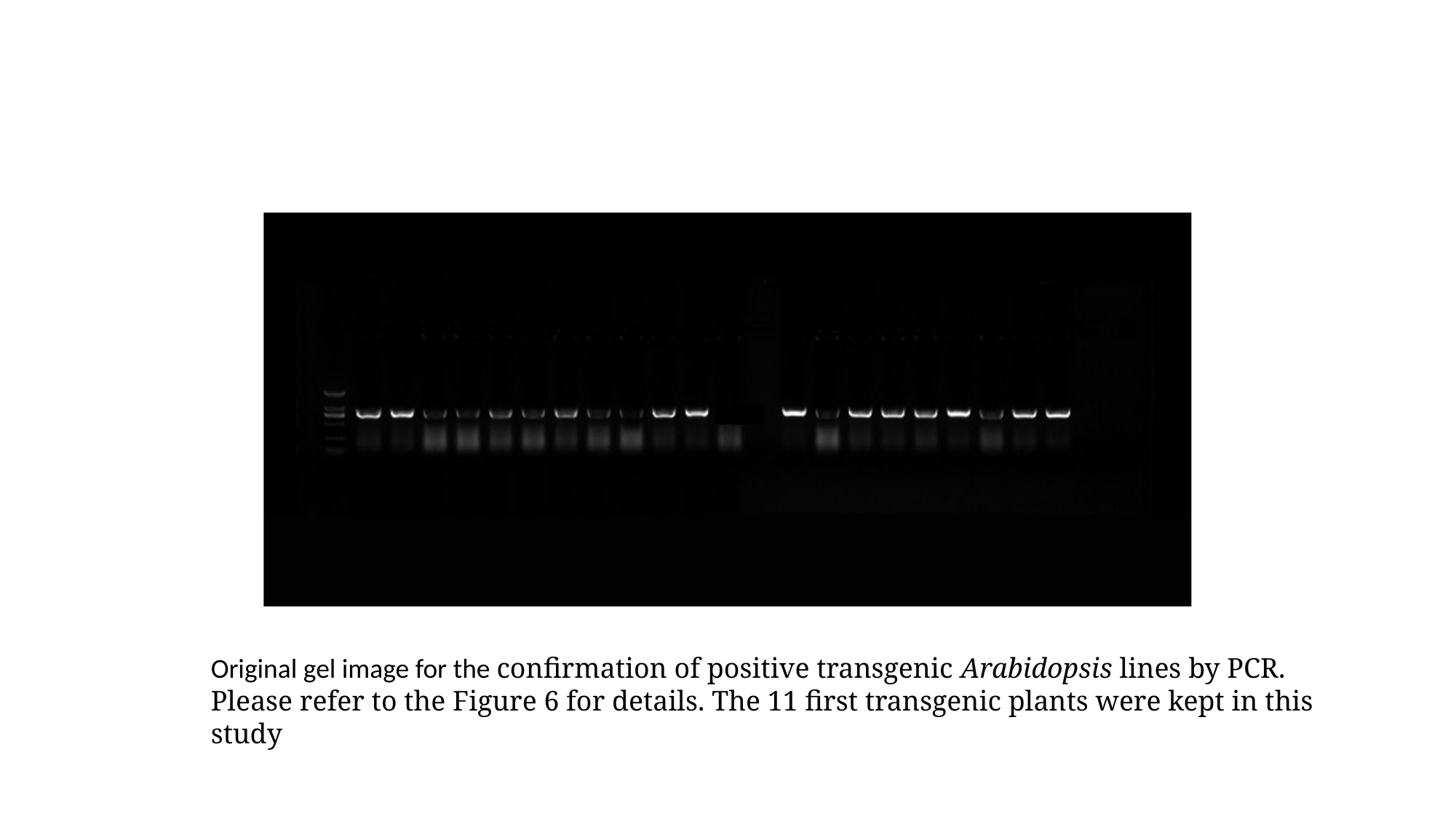

Original gel image for the confirmation of positive transgenic Arabidopsis lines by PCR. Please refer to the Figure 6 for details. The 11 first transgenic plants were kept in this study

## Slide 3
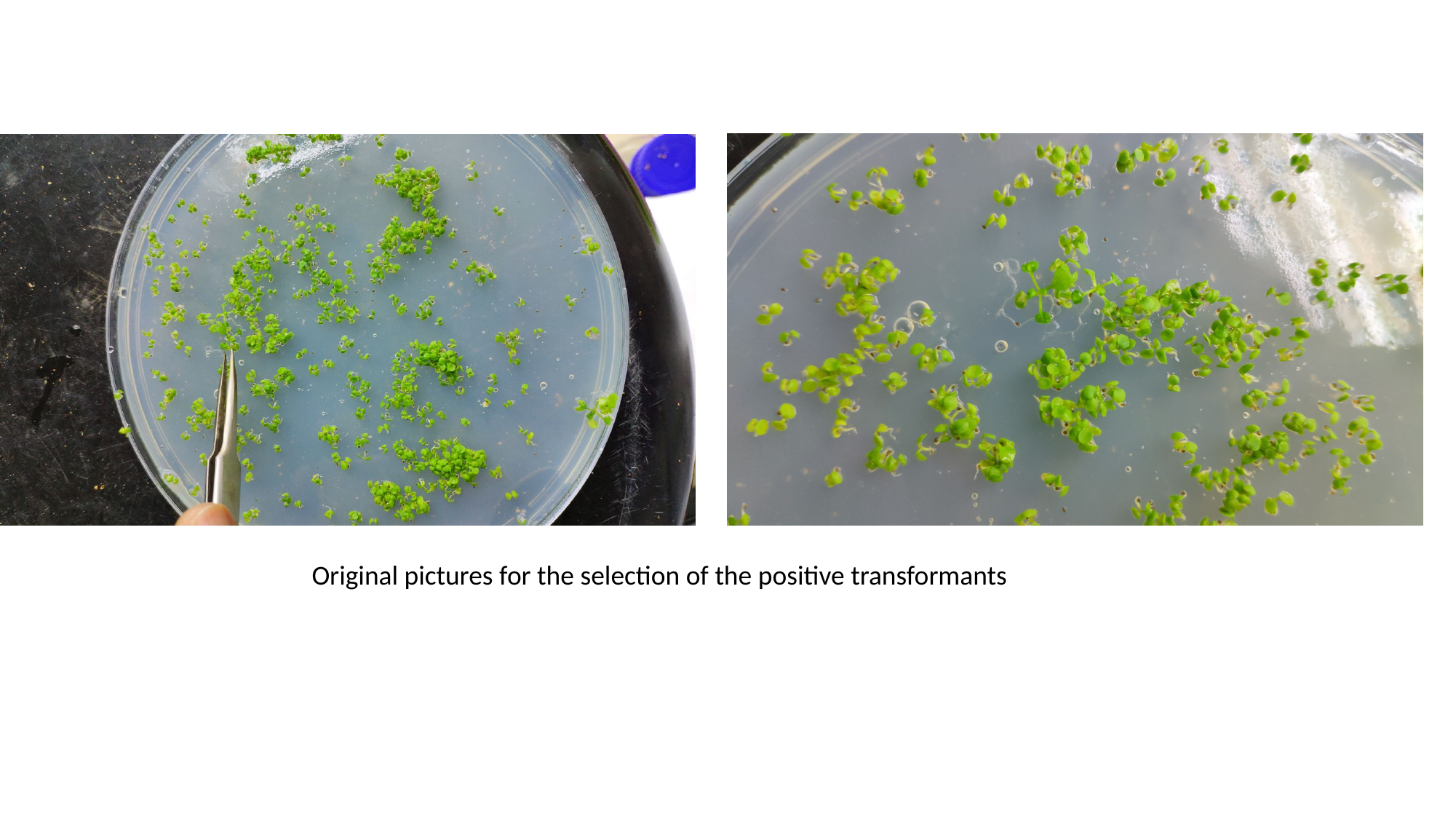

#
Original pictures for the selection of the positive transformants

## Slide 4
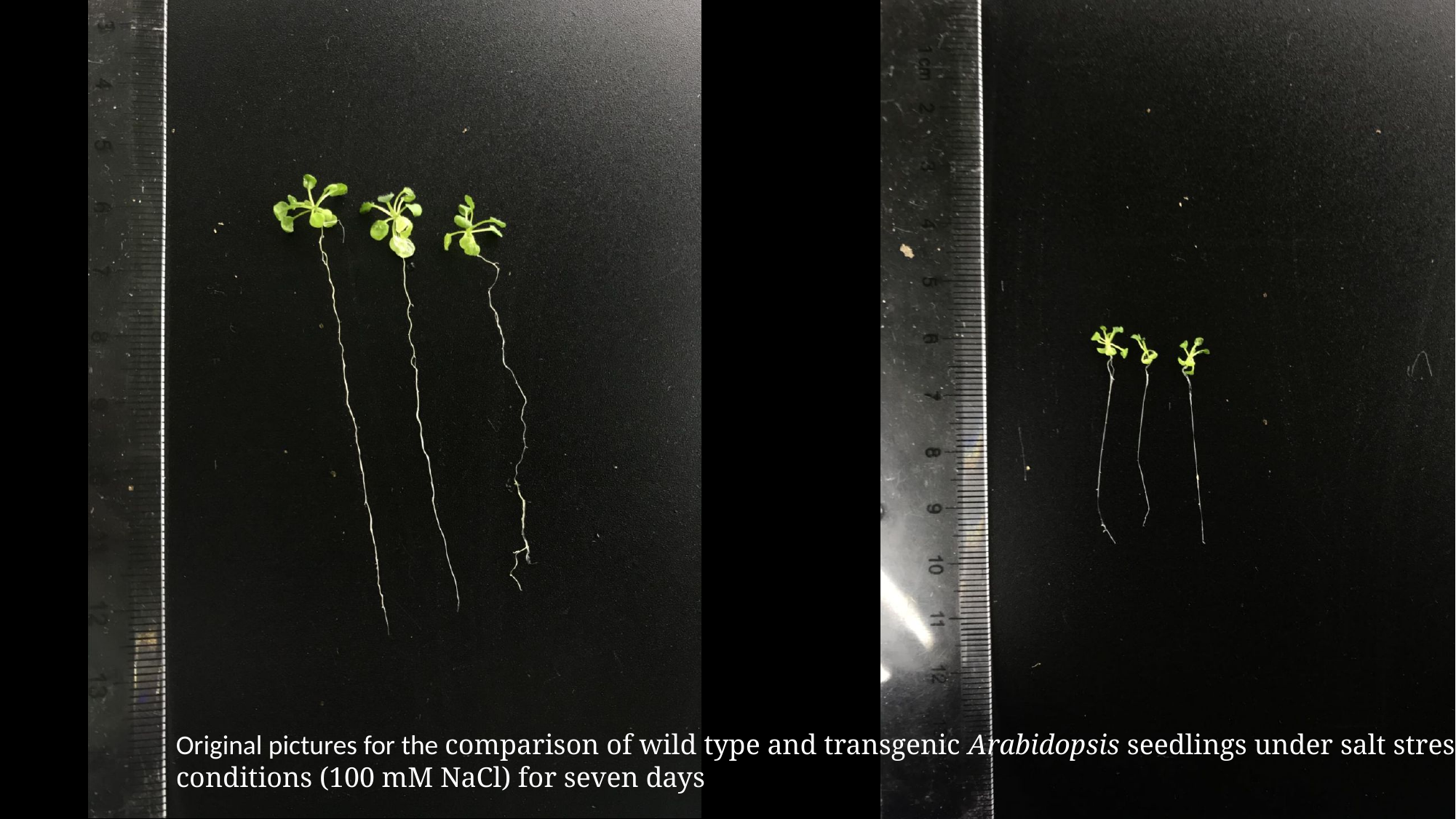

Original pictures for the comparison of wild type and transgenic Arabidopsis seedlings under salt stress conditions (100 mM NaCl) for seven days
