# Supplementary material for: Transcriptome analysis reveals the key pathways and candidate genes involved in salt stress responses in Cymbidium ensifolium leaves
Source: BMC Plant Biol. 2023 Feb 1;23:64. doi: 10.1186/s12870-023-04050-z (PMC9890885; doi:10.1186/s12870-023-04050-z)
Supplement: Supplementary file 3 — Additional file 3: Supplementarydata 1. Original pictures used to prepare the Figure 6 [file 12870_2023_4050_MOESM3_ESM.pptx]

## Slide 1
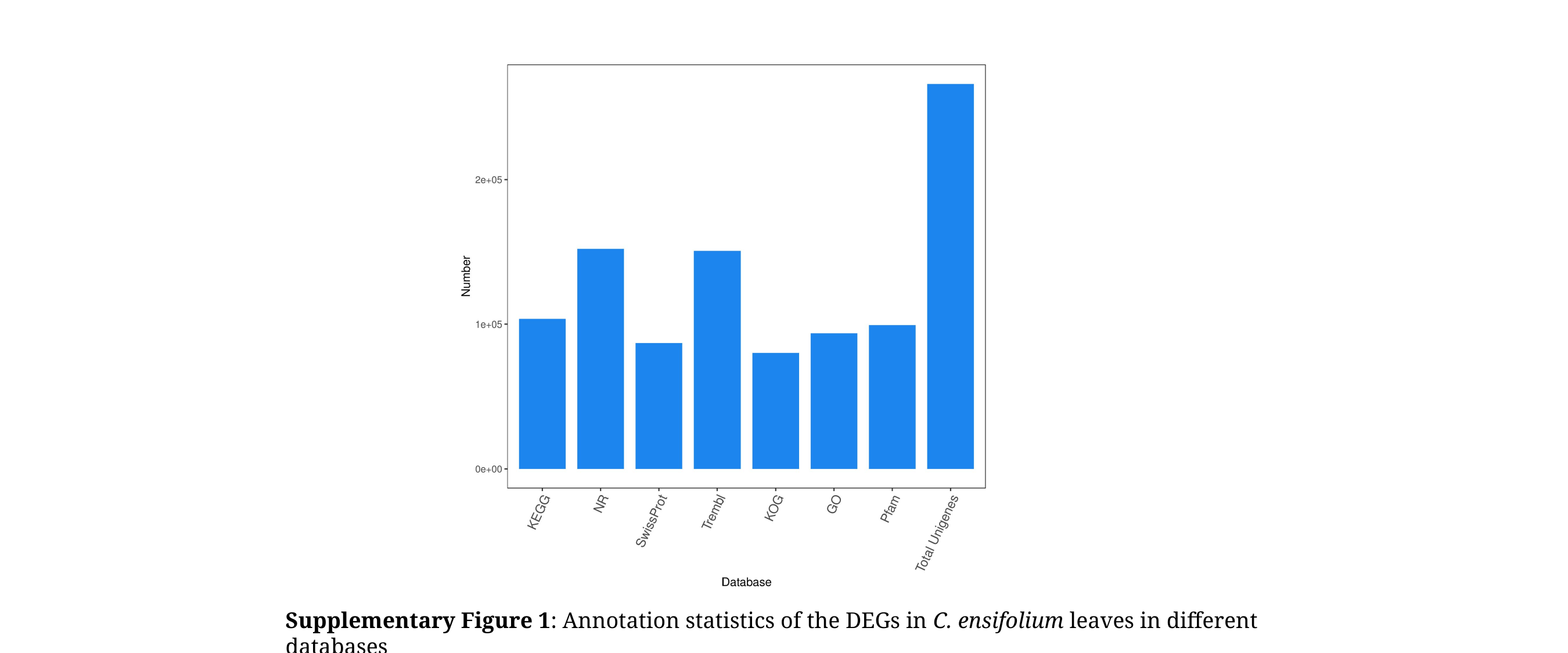

Supplementary Figure 1: Annotation statistics of the DEGs in C. ensifolium leaves in different databases
